# Comparative Transcriptome Analysis of SE initial dedifferentiation in cotton of different SE capability

**DOI:** 10.1038/s41598-017-08763-8

**Published:** 2017-08-17

**Authors:** Aiping Cao, Yinying Zheng, Yu Yu, Xuwen Wang, Dongnan Shao, Jie Sun, Baiming Cui

**Affiliations:** 1 College of Agriculture/The Key Laboratory of Oasis Eco-Agriculture, Shihezi University, Shihezi, China; 2 Colleges of Life Science, Shihezi University, Shihezi, China; 3 Cotton research Institute, XinJiang Academy of Agricultural and Reclamation Science, Shihezi, China

## Abstract

Somatic embryogenesis (SE) is a critical transition from vegetative to embryogenic growth in higher plants; however, few studies have investigated the mechanism that regulates SE initial differentiation. Most cotton varieties have not undergone regeneration by SE, so only a few varieties can be used in genetic engineering. Here, two varieties of cotton with different SE capabilities (HD, higher differentiation and LD, lower differentiation) were analyzed by high throughout RNA-Seq at the pre-induction stage (0h) and two induction stages (3h and 3d) under callus-induction medium (CIM). About 1150 million clean reads were obtained from 98.21% raw data. Transcriptomic analysis revealed that “protein kinase activity” and “oxidoreductase activity” were highly represented GO terms during the same and different treatment stages among HD and LD. Moreover, several stress-related transcription factors might play important roles in SE initiation. The SE-related regulation genes (*SERKs*) showed different expression patterns between HD and LD. Furthermore, the complex auxin and ethylene signaling pathway contributes to initiation of differentiation in SE. Thus, our RNA-sequencing of comparative transcriptome analysis will lay a foundation for future studies to better define early somatic formation in cotton with different SE capabilities.

## Introduction

Somatic embryogenesis (SE) resulting in regeneration of new plants is an important step in the Agrobacterium-mediated method. This process includes two stages. First, the cell reverts back to its own lineage to re-enter the cell cycle and transform into a dedifferentiated cell state. Callus formation is frequently considered the manifestation of the dedifferentiated cellular state. Second, calli can regenerate somatic embryos and new plants by redifferentiation^1, 2^. Dedifferentiation is frequently considered the manifestation of the stem cell-like state to switch fate preceding the commitment for proliferation^3^. Following that, dedifferentiation is an important biological phenomenon in whole SE process.

Various molecular biological technologies have been described to investigate molecular mechanisms during SE in different plant species^4^, including carrot^5^, *Arabidopsis* ^2^, soybean^6^ and potato^7^. These studies have revealed valuable information regarding the physiological and biochemical changes that occur during SE^8^. In addition, many studies have investigated the initiation of SE in various plant species. Plant hormones and stress are the two most important factors involved in stimulation of SE initiation^9^. However, the regulatory mechanism of vegetative-to-embryogenic triggered remains elusive^10^. For example, numerous candidate genes associated with hormonal regulation, oxidative stress and response to stress were shown to be specifically activated or exhibit differential expression^11, 12^.

Cotton is a major raw material used by the textile industry and a source of oil. Transgenic technologies have been widely applied to cotton molecular breeding through Agrobacterium-mediated transformation via SE. However, few varieties have been successfully used in genetic engineering and regeneration *in vitro* ^13^. The majority of the desirable cotton cultivars are incapable of regeneration via SE^14^, and many factors can effect SE, including culture conditions and tissue background, furthermore, the key factor is a genotype-dependent response^15^. Several recent studies have been conducted to investigate the molecular mechanism and identify genes critical for SE^16^. More recently, RNA deep-sequencing technology has provided a platform for analysis of differences in gene expression^17^. There are reports demonstrating that hormonal signaling pathways such as auxin and cytokinin^16^, as well as stress-responsive pathways are critical during cotton SE development^18^. However, the initial SE molecular mechanisms have not been thoroughly studied and are still unclear in different dedifferentiated cotton cultivars. Here, we investigated early events of SE during the without induction stage (0h) and induction stage (3h and 3 d) by comparing cotton of different SE capability (HD and LD) using RNA-seq profiling combined with differentially expressed genes (DEG) and GO analysis. This study provides a comprehensive analysis of gene expression of different cotton types during the initial stages of somatic embryogenesis. Furthermore, the data presented herein provides an abundant gene catalog that should be a useful resource for future studies of molecular and developmental mechanisms of SE.

## Results

### Differentiation efficiency of various cotton species

**Figure 1:**
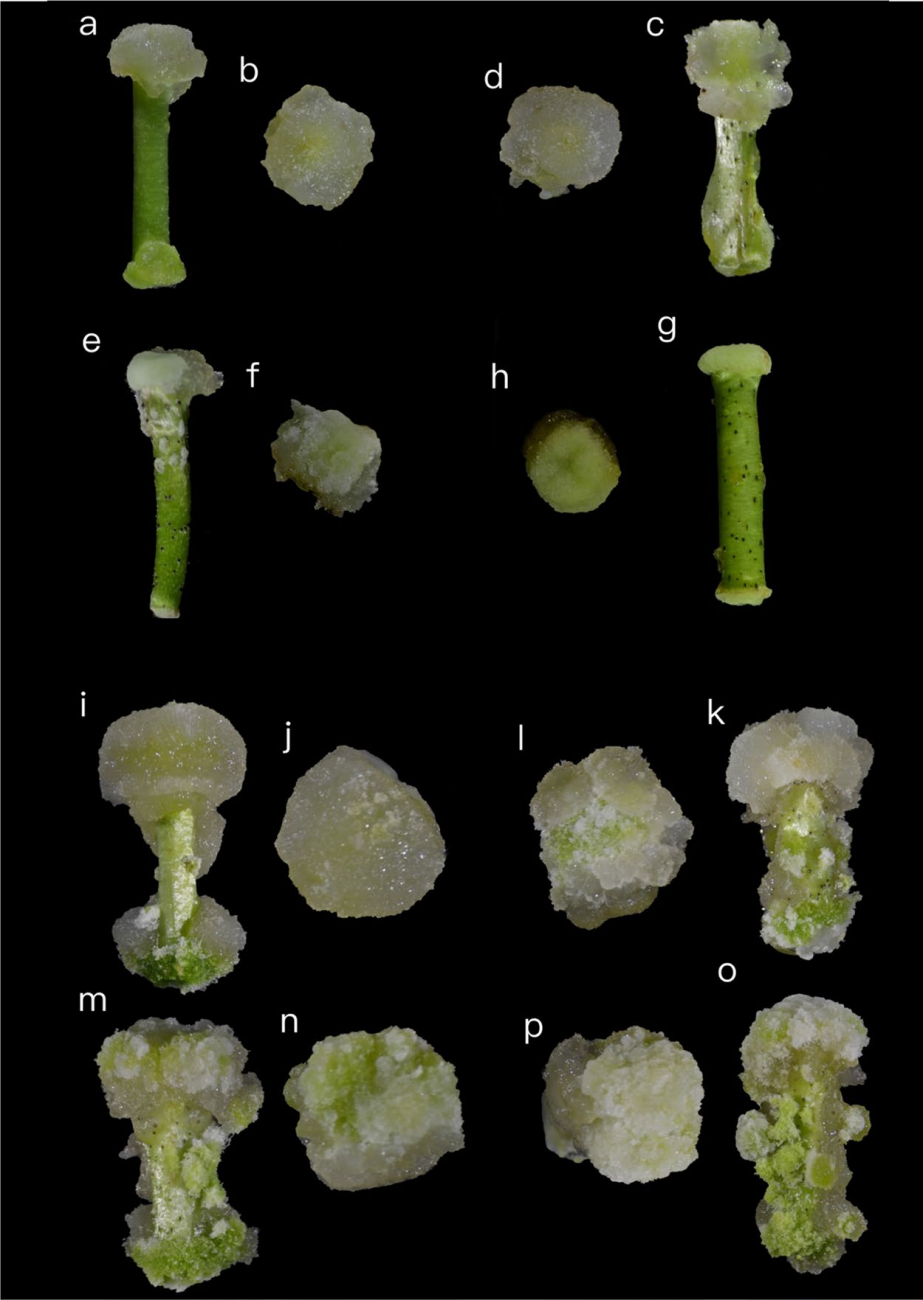
Four-cotton cultivars showing different differentiation at 10d and 1 month in the CIM. (**a–g**) after 10d of culture in CIM. (**i–o**) after 1M of culture in CIM. Four cotton cultivars of different induction stages were sampled, YZ1 (**a**,**b**,**i** and **j**), R15 (**c**,**d**,**k** and **l**), X33 (**e**,**f**,**m** and **n**) and X42 (**g**,**h**,**o** and **p**).

Four *Gossypium hirsutum L* cultivars (YZ1, R15, X33 and X42) were sampled at three stages, the pre-induction stage (0h) and two induction stages (3h and 3d) in the CIM. YZ1 and R15 (HD) were selected because they exhibited good SE potential and were the main transgenic materials. Although X33 and X42 (LD) are the major commercial cultivars in Xinjiang, China, they have a low rate of differentiation during SE relative to YZ1 and R15. The high differentiation lines and the recalcitrant lines showed diverse morphology during the callus induction stage. The early calli of HD were friable, loose, and primarily whitish (Fig. 1a–d and i–l), while those of LD were firmer and less friable than HD (Fig. 1e–g and m–p). The significant differences in calli may be due to differences in gene expression during SE initiation; therefore, cDNA libraries were constructed from three time stages between HD and LD and two biological replicates were performed.

### Illumina HiSeq mRNA sequencing

High-throughput RNA-Seq sequencing generated 1171 million raw reads from 24 RNA samples using the Illumina HiSeqTM 2000 platform, approximately 48 million reads for each line. After discarding adapters, poly-N and low quality reads from raw data, a total of 1150 million clean reads were obtained from the total raw reads (98.26%). Two biological replicates were evaluated for each line, and the results indicated good agreement of the RNA-seq data (0.958 < R2 < 0.986). Overall, 86.31%–88.94% of the high-quality reads were mapped to the *Gossypium hirsutum* reference genome using TopHat29. Of the mapped reads, 84.3–89.4% were distributed in exon regions, while 1.7–2.4% were located in introns and 8.0–13.2% were in intergenic regions (Table 1). For all sequence data, the average Q20, Q30, and GC contents were 97.03%, 92.64%, and 43.82%, respectively. The error rate of all samples was 0.01%–0.02% (Table. S1). Of which three categories of mapped data: 1) multiple mapped (7.63–9.11%) and unique mapped reads (77.75–81.06%), 2) forward mapped (38.83–40.53%) and reverse mapped reads (38.88–40.54%), and 3) non-splice reads (48.35–53.15%) and splice reads (26.87–29.64%) (Table S1).

**Table 1:** Summary of transcriptome sequencing data.

Sample name	Raw reads	Clean reads	Clean bases	Total mapped	Exon (%)	Intron (%)	Intergenic (%)
R15_0h_1	45813740	44974414	6.75 G	38815533	84.3	2.4	13.2
R15_0h_2	44436266	43634922	6.55 G	38509898	85.7	2.2	12
R15_3h_1	46339512	45602268	6.84 G	40352472	87.6	1.8	10.6
R15_3h_2	55664292	54795000	8.22 G	48396413	88.2	1.8	10
R15_3d_1	58297408	57185824	8.58 G	49594358	87.2	1.9	10.9
R15_3d_2	45504132	44718654	6.71 G	39092086	86.4	1.8	11.8
YZ1_0h_1	54411212	53434048	8.02 G	46862756	87	2	11
YZ1_0h_2	42332338	41472822	6.22 G	36244862	87.2	2	10.8
YZ1_3h_1	48182224	47414878	7.11 G	41720626	88.2	1.8	10
YZ1_3h_2	51910688	51103964	7.67 G	45108556	89.4	1.7	8
YZ1_3d_1	41334482	40551474	6.08 G	35085465	88	1.8	10,2
YZ1_3d_2	53111840	51931008	7.79 G	45087397	87.9	1.7	10.3
X33_0h_1	55528574	54606234	8.19 G	48091726	85.1	2.3	12.5
X33_0h_2	48595188	47799278	7.17 G	42052352	86.5	2	11.5
X33_3h_1	51455830	50559176	7.58 G	44749580	87.9	1.8	10.2
X33_3h_2	47675942	46858792	7.03 G	41408584	87.8	1.7	10.5
X33_3d_1	44247472	43462830	6.52 G	38118346	87.7	1.7	10.6
X33_3d_2	45522790	44759502	6.71 G	39403962	86.8	1.8	11.3
X42_0h_1	40809328	40079772	6.01 G	35489334	86.9	1.9	11.2
X42_0h_2	49641394	48808912	7.32 G	43159067	86.6	2.1	11.3
X42_3h_1	49429384	48648396	7.3 G	43221078	88.5	1.9	9.7
X42_3h_2	45973326	45219188	6.78 G	40215890	87.3	2	10.7
X42_3d_1	55203894	54213944	8.13 G	47493700	86.3	2	11.7
X42_3d_2	49845162	49014372	7.35 G	43117216	87	1.9	11.1

### DEGs and GO terms during same-stage comparisons

**Figure 2:**
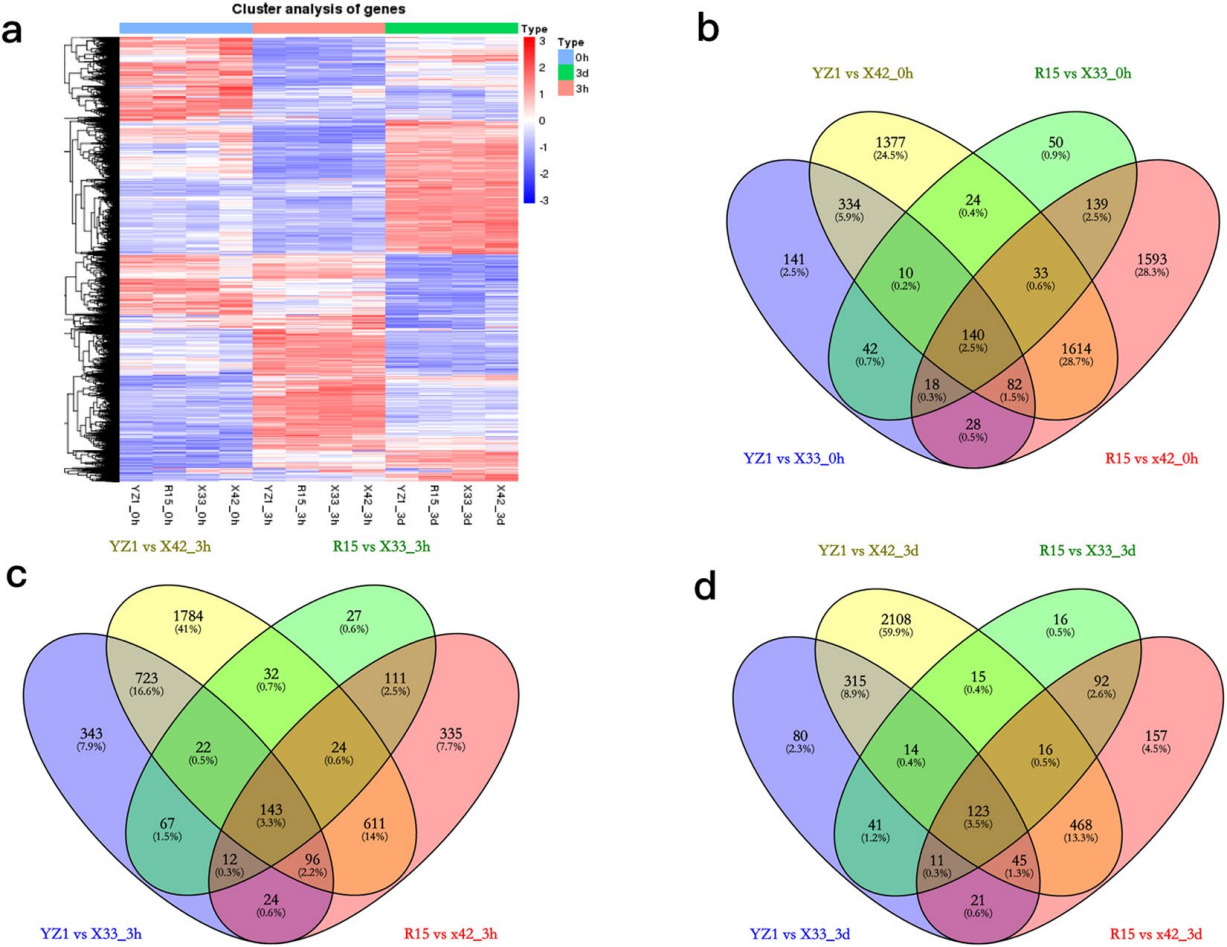
DEGs in three same-stage comparisons between HD and LD. (**a**) Heatmap of DEGs (P-value < 0.05, |log2FoldChange| > 2 at one sampling point); (**b**–**d**) Venn analysis of the DEGs in two types of cotton during three time stages.

Significant differences in gene expression were examined in three same-stage comparisons of the two difference species (HD and LD). Using a P-value < 0.05 and |log2FoldChange| > 2 in one sampling point as the higher criteria. Our date shows a heatmap of DEGs and Venn diagrams showing details regarding the number of DEGs at each pairwise comparison (Fig. 2). The results revealed 140 DEGs at 0h (Fig. 2b), 143 DEGs at 3h (Fig. 2c) and 123 DEGs at 3d (Fig. 2d). Analysis of these DEGs revealed that protein kinase-related genes were differentially expressed. The 0h comparison led to identification of one G-type lectin S-receptor-like Serine/Threonine-kinase and three putative homologs of the protein kinase superfamily protein. Additionally, two homologs of the G-type lectin S-receptor-like Serine/Threonine-kinase, two protein kinase superfamily proteins and one MAP kinase kinase 2 were involved at 3h. In addition, the G-type lectin S-receptor-like Serine/Threonine-kinase, protein kinase superfamily protein and MAP kinase kinase 2 were significantly down-regulated at 3d (Table 2).

**Table 2:** Protein kinase-related genes differentially expressed in the same-stage.

	Gene id	log2 Fold Change				Gene description
		R15 vs X33	R15 vs X42	YZ1 vs X33	YZ1 vs X42	
0h	Gh_A06G1786	−3.6825	−3.2886	−3.9907	−3.5972	G-type lectin S-receptor-like Serine/Threonine-kinase
	Gh_A03G1310	2.0274	1.1129	1.989	1.0746	Protein kinase superfamily protein
	Gh_A06G1617	−3.2889	−2.0637	−2.715	−1.489	
	Gh_A11G2003	1.3657	1.7908	1.5901	2.0144	
	Gh_A02G1701	1.2525	1.3822	1.3948	1.5242	
3h	Gh_A06G1786	−2.6061	−2.6257	−3.5518	−3.5623	G-type lectin S-receptor-like Serine/Threonine-kinase
	Gh_D10G0162	2.2204	2.5952	2.4067	2.7904	
	Gh_A06G1617	−5.1736	−3.7709	−4.0353	−2.6227	Protein kinase superfamily protein
	Gh_A11G2002	−2.3912	−2.7061	−1.3735	−1.6803	
	Gh_D07G2384	−2.0518	−2.1876	−1.6726	−1.7997	MAP kinase kinase 2
3d	Gh_A06G1786	−2.8407	−3.143	−2.859	−3.1611	G-type lectin S-receptor-like Serine/Threonine-kinase
	Gh_A06G1617	−2.5474	−1.322	−2.6999	−1.4741	Protein kinase superfamily protein
	Gh_D07G2384	−2.7462	−2.4769	−1.8776	−1.6079	MAP kinase kinase 2

### Comparison of DEGs and GO terms at different-stages

**Figure 3:**
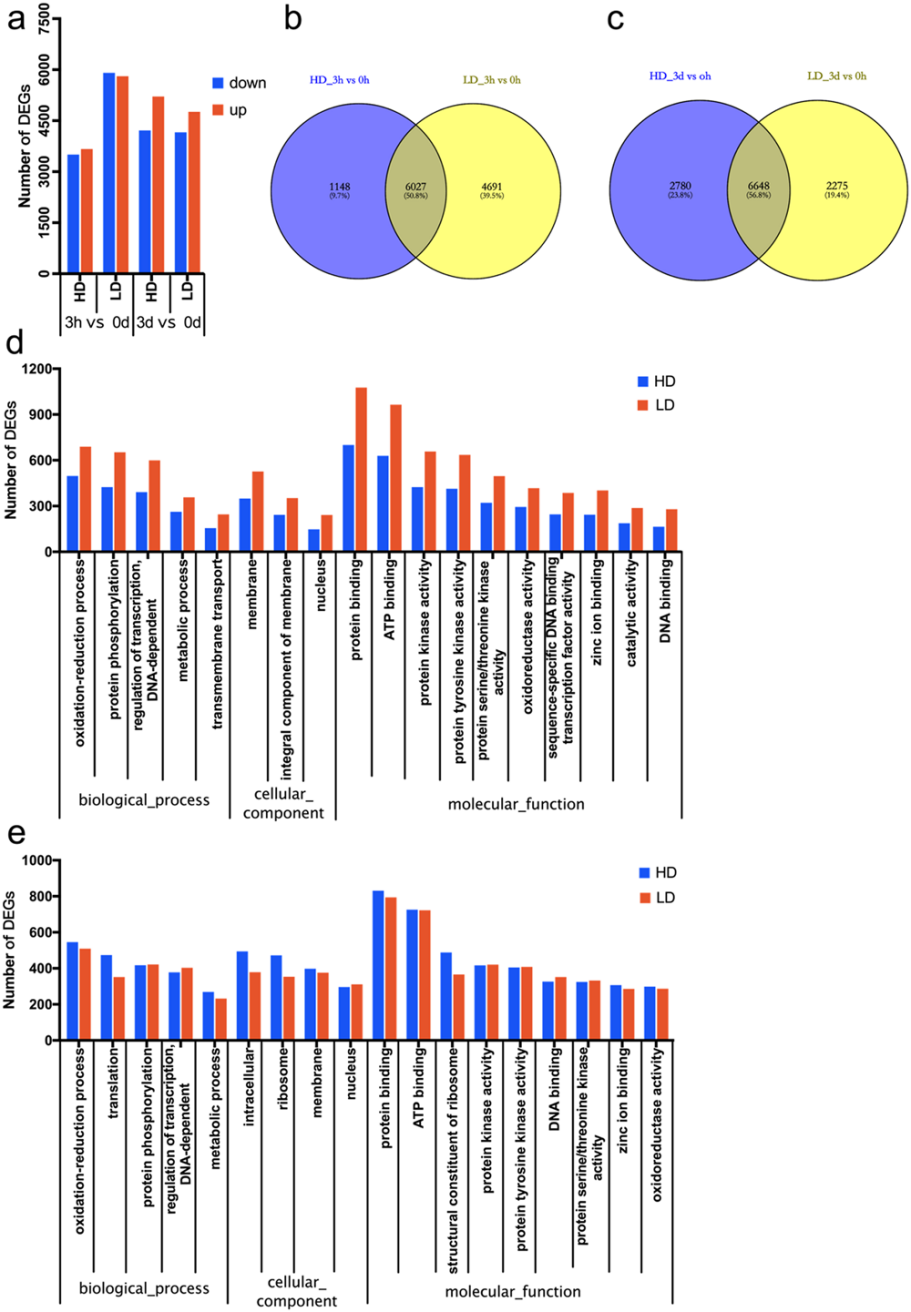
DEGs in four cotton cultivars during different-stage comparisons. (**a**) Number of DEGs at 3h vs 0h and 3d vs 0h; (**b**,**c**) Venn analysis of the DEGs in two types of cotton during different comparisons. (**d**,**e**) Go function analysis of DEGs at 3h vs 0h and 3d vs 0h, respectively.

DEGs and three main GO terms were identified by comparisons of two induction stages (3h and 3d) and the pre-induction stage (0h). Comparison of 3h vs 0h revealed 7175 and 10,718 DEGs in the HD and LD cultivars, respectively, and more than half (50.8%) of the DEGs were present in all cotton cultivars at 3h vs 0h (Fig. 3a and b). Upon GO classification based on biological processes, “oxidation-reduction process”, “protein phosphorylation” and “regulation of transcription, DNA-dependent” were found to be associated with a higher number of DEGs in HD and LD (Fig. 3d). The six top molecular functions were “protein binding”, “ATP binding”, “protein kinase activity”, “protein tyrosine kinase activity”, “protein serine/threonine kinase activity” and “oxidoreductase activity” (Fig. 3d). Among cellular components, “membrane”, “integral component of membrane” and “nucleus” were highly represented in both cotton types (Fig. 3d).

Comparison of the 3d vs 0h samples revealed 9428 DEGs in the HD species and 8923 DEGs in the LD cultivars (Fig. 3a and c). “Oxidation-reduction process”, “translation” and “protein phosphorylation” were the main GO terms identified among differentially expressed genes corresponding to biological processes. Among molecular functions, the highly represented GO terms were “protein binding”, “ATP binding”, “structural constituent of ribosome”, “protein kinase activity”, “protein tyrosine kinase activity” and “DNA binding”. “Intracellular”, “ribosome” and “membrane” were grouped into cellular components in HD and LD (Fig. 3e).

The number of DEGs identified in LD was higher than in HD at 3h vs 0h, while the number of DEG detected at 3d vs 0h showed the opposite pattern. With the exception of the comparisons of LD at 3h vs 0h, the number of up-regulated DEGs was slightly higher than that of down-regulated DEGs in both comparisons (Fig. 3a). The cellular component was the most overrepresented GO term in different-stage comparisons between HD and LD.

### Analysis of TF during SE initiation

**Figure 4:**
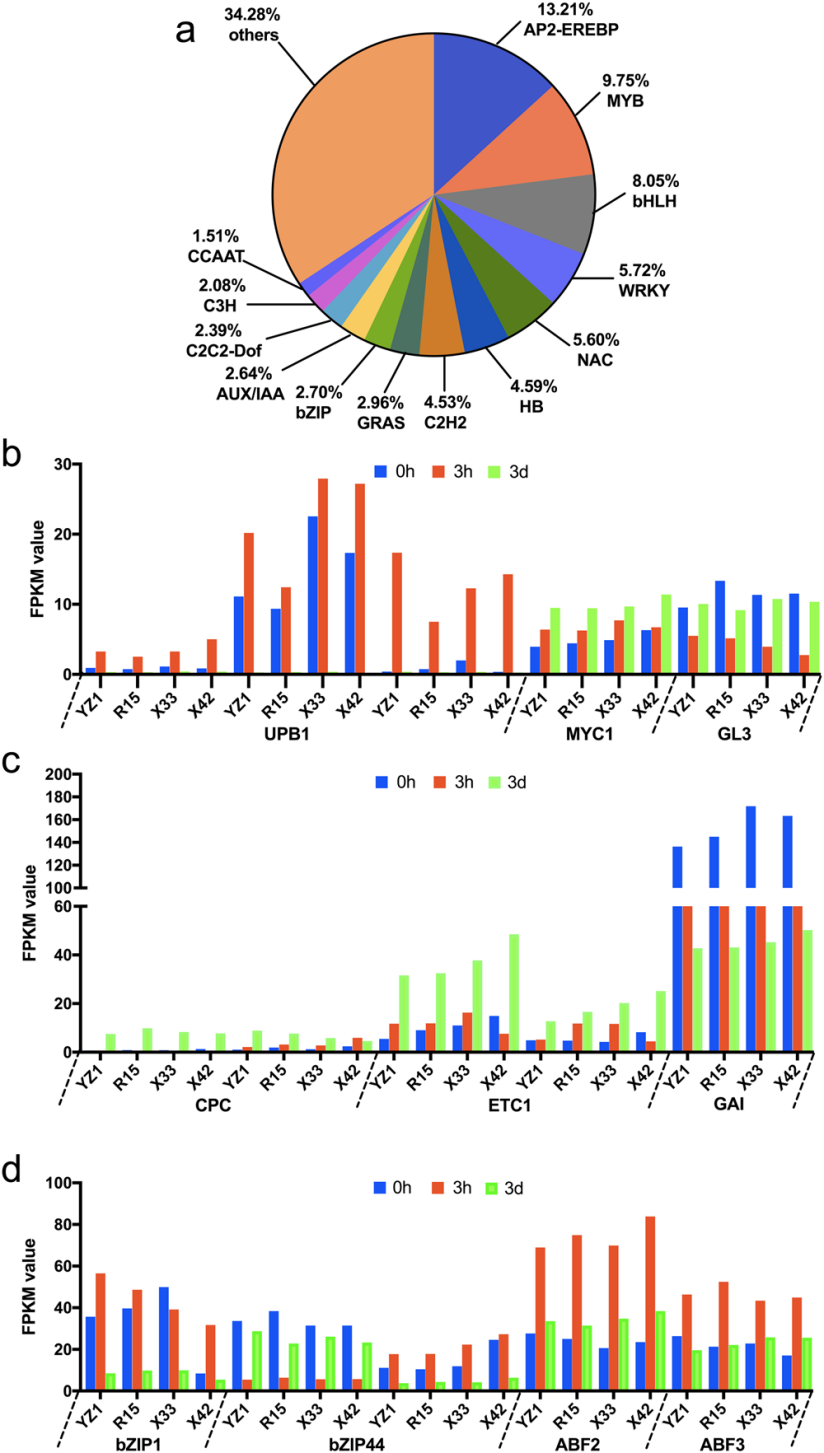
(**a**) TF genes and classification of TF families. TF genes were classified into TF families using iTAK. A total of 1590 different transcription factors were annotated in 69 TF families. Numbers represent the percentage of TF genes. (**b**–**d**) Detailed expression profiles of SE differentiation related TF genes. The x-axis indicates the distribution of SE differentiation related TF genes in the four cotton species, and the y-axis represents the value of FPKM in each TF gene.

Transcription factors (TFs) recognize DNA in a sequence-specific manner to regulate gene expression. Based on the |log2FoldChange| > 2 at one sampling point, a total of 1590 specifically different transcription factors were annotated in 69 TF families between two cultivars. Most TFs were involved in response to pathogen challenge, including AP2-EREBP (211), MYB (155), bHLH (128), WRKY (91), NAC (89), HB (73), C2H2 (72), GRAS (47), bZIP (43), AUX/IAA (42), C2C2-Dof (38), CH3 (33) and CCAAT (24) (Fig. 4a). Putative homologs of SE differentiation related genes were identified in two cotton cultivars (Fig. 4b–d). *UPBEAT1* (*UPB1*), belongs to bHLH TF family, three *UPB1* homologues were same expression model and were up-regulated during at 3h, while then the expression level reduced to very low (FPKM < 1) at 3d. However, another two bHLH transcription factors, *MYC1* and *GL3*, showed higher expression levels at 3d. The expression of *MYC1* homologues increased with induction. *GL3* decreased at 3h, then increased at 3d. The MYB transcription factors *CPC* and *ETC1* showed different expression patterns during three time stages. *GAI* is a subfamily of the *GRAS* family, the value of FPKM was decreased with induction. bZIP transcription factors, *bZIP1*, *bZIP44* and acid responsive elements-binding factor, *ABF2* and *ABF3*, most of those TFs showed higher FPKM at the 3h, while *bZIP44* (Gh_A06G1847) was generally opposite expression pattern.

### Analysis of SE-related functional genes

**Figure 5:**
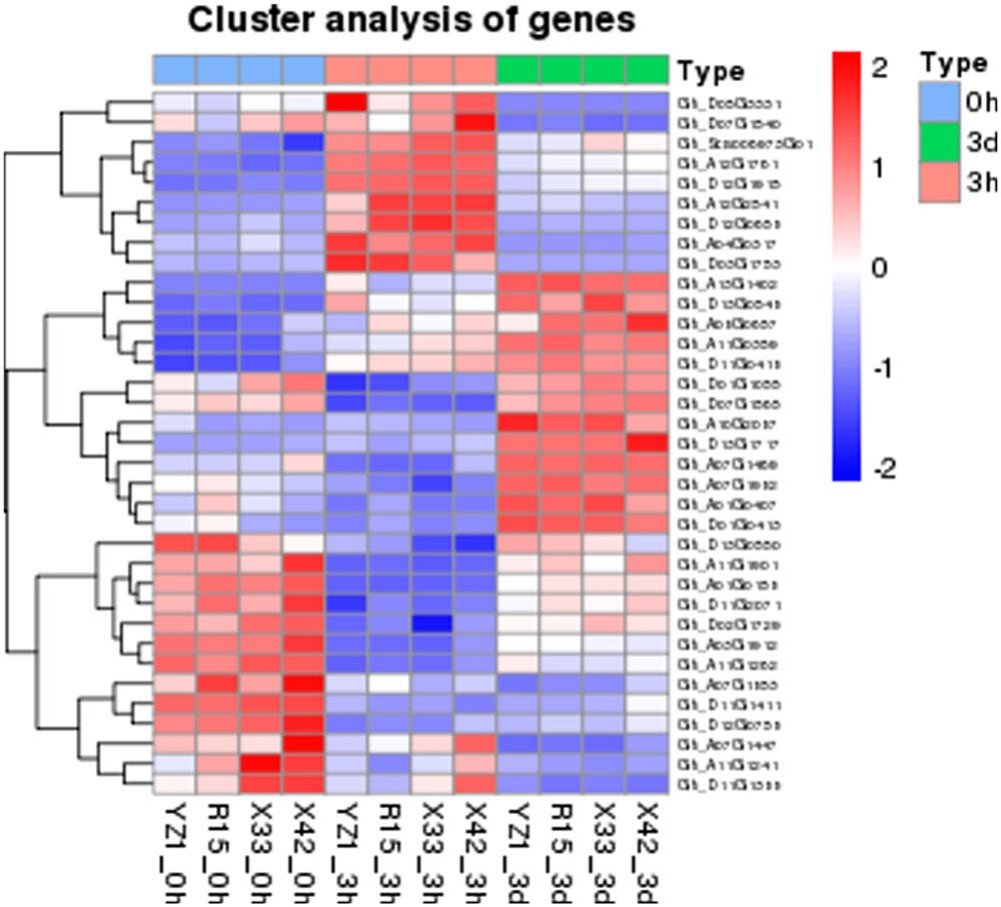
Heatmap of SE-related functional genes (P-value < 0.05, |log2FoldChange| >2 at one sampling point).

A number of embryogenic-regulating genes that were differentially expressed during early SE (|log2FoldChange| > 2 at one sampling point) were selectively analyzed. The expression patterns of SE-related genes are shown in Table 3 and Fig. 5. *WUSCHEL*-related homeobox genes were involved, including *WOX5* (1) and *WOX11* (2). Overall, *WOX* homeobox genes were significantly up-regulated in the two cotton cultivars at 3d vs 0h, while there was no significant difference in expression between 3h and 0h. *WOX5* and *WOX11* exhibited similar expression patterns at 3d vs 0h, which were just significant up-regulated in HD and LD. In the present study, *SERK1* (somatic embryogenesis receptor kinase 1), *SERK2* (somatic embryogenesis receptor kinase 2), *BAK1* (*SERK3/BRI1*-associated kinase 1), *TDR/PXY* (TDIF receptor/phloem intercalated with xylem) and *BAM1* were identified in two cotton types. *SERK1* showed a similar expression pattern at 3h vs 0h between HD and LD. *SERK2* and one *BAK1* were up-regulated at 3d vs 0h, while *BAK1* displayed the opposite expression pattern in LD. Four homologs of *BAM1* were found to be down-regulated at 3h vs 0h, while just one *BAK1* (Gh_D12G0758) was down-regulated at 3d vs 0h among all cotton cultivars. The *CLAVATA3/ESR-RELATED* (*CLE*) family of seven genes was showed, these genes involving *CLE1*, *CLE5*, *CLE27*, *CLE44* and *TDIF*. At 3h vs 0h, *CLE1* and *CLE27* showed the opposite expression in LD cotton, while *CLE44* and two *TDIF* were down-regulated between HD and LD. At 3d vs 0h, *CLE44* and one *TDIF* were down-regulated in both cultivars, while *CLE5* (Gh_D01G0413) and *TDIF* (Gh_A07G1469) were up-regulated in LD and HD, respectively. Five homologous genes of EBE were significantly upregulated at 3h compared to 0h in all cotton cultivars. With the exception of Gh_A11G0359 and Gh_A12G2541, the three *EBE* homologous genes were also up-regulated at 3d vs 0h in the two cotton cultivars, while one *EBE* (Gh_A11G0309) was up regulated at 3d vs 0h in the HD. One *YUC4*, four *YUC8* and one *YUC10* were confirmed to show differential expression patterns between cotton types. Only *YUC4* was up-regulated in the HD cultivars at 3h vs 0h. At 3d vs 0h, *YUC8* and *YUC10* showed the opposite expression patterns in the two types.

**Table 3:** The log2 fold change of SE-related genes in different-stage comparisons.

Gene name	Gene ID	log2 Fold Change							
		3h vs 0h				3d vs 0h			
		YZ1	R15	X33	X42	YZ1	R15	X33	X42
WOX5	Gh_A10G2087	None	None	None	None	3.08	Inf	5.45	Inf
WOX11	Gh_A13G1402	None	None	None	None	Inf	Inf	Inf	Inf
WOX11	Gh_D13G1717	None	None	None	None	Inf	Inf	Inf	6.17
SERK1	Gh_A01G0158	−3.69	−4.36	−4.09	−4.51	None	None	None	None
SERK2	Gh_Sca006973G01	None	None	2.11	2.63	None	None	None	None
BAK1	Gh_D13G0548	None	None	2.32	2.56	None	None	None	None
BAK1	Gh_D13G0550	None	None	−2.35	−2.35	None	None	None	None
TDR/PXY	Gh_A06G0865	−2.79	−2.44	−2.64	−3.21	None	None	None	None
BAM1	Gh_A11G1901	−4.28	−3.49	−4.30	−4.65	None	None	None	None
BAM1	Gh_D12G0758	−3.23	−2.99	−3.29	−2.97	−2.38	−2.18	−2.60	−2.70
BAM1	Gh_D11G2071	−2.69	−2.14	−2.06	−2.57	None	None	None	None
BAM1	Gh_A03G1912	−3.61	−3.49	−3.75	−3.07	None	None	None	None
TDIF	Gh_A07G1469	None	None	None	None	3.38	3.40	None	None
TDIF	Gh_D07G1565	−2.27	−2.04	−2.11	−2.66	None	None	None	None
TDIF	Gh_D11G1411	−3.22	−3.78	−3.88	−4.79	−3.76	−3.66	−3.82	−2.67
CLE1	Gh_A04G0317	None	None	3.25	5.10	None	None	None	None
CLE5	Gh_D01G0413	None	None	None	None	None	None	4.18	4.54
CLE27	Gh_D02G1729	None	None	−4.83	−2.35	None	None	None	None
CLE44	Gh_A11G1262	−6.00	−4.66	−5.76	−4.44	−2.19	−2.75	−3.33	−2.78
YUC4	Gh_D03G1753	5.06	Inf	None	None	None	None	None	None
YUC8	Gh_A07G1853	None	None	None	None	−3.90	−3.95	None	None
YUC8	Gh_D07G1540	None	None	None	None	None	None	−4.91	−4.53
YUC8	Gh_A11G1241	None	None	None	None	None	None	−4.99	−3.36
YUC8	Gh_D11G1388	None	None	None	None	None	None	−4.26	−5.63
YUC10	Gh_A08G0657	None	None	None	None	2.27	3.60	3.06	2.35
EBE	Gh_D12G0658	6.08	7.37	5.89	7.27	None	None	None	None
EBE	Gh_D11G0418	3.98	3.97	3.84	3.03	5.21	5.02	4.52	3.20
EBE	Gh_A12G1761	5.71	6.26	7.90	6.84	2.52	3.16	4.41	3.83
EBE	Gh_A12G2541	3.66	6.14	6.07	5.97	None	None	None	None
EBE	Gh_D12G1915	6.19	6.33	6.03	6.25	2.33	2.97	2.45	2.81
EBE	Gh_A11G0359	None	None	None	None	3.00	2.88	None	None

(Note: Among the 3d vs 0h, Inf indicates that the FPKM of WOX5 was close to 0 at 3d).

### Validation of RNA-seq data by qRT-PCR

**Figure 6:**
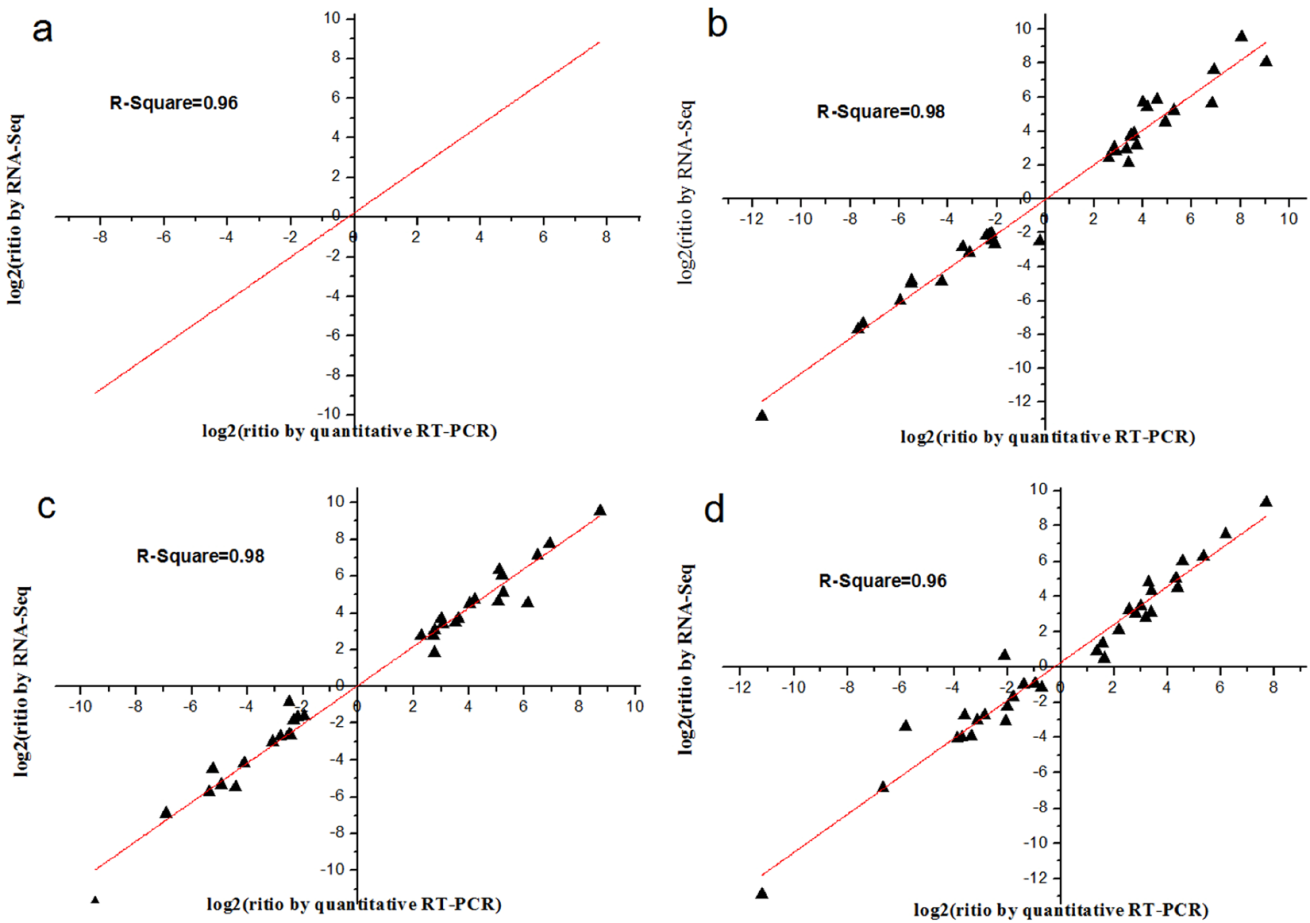
Comparison of expression profile by RNA-Seq and qRT-PCR. (**a**–**d**) Comparison of expression profile of 11 randomly selected DEGs by RNA-Seq and qRT-PCR at YZ1, R15, X33 and X42 (3h vs 0h, 3d vs 0h and 3d vs 3h).

To further validate the RNA-seq data, 11 DEGs were randomly selected along with their specific primers for qRT-PCR analysis. The cotton *ERF1α* (NCBI Reference Sequence: XM_016892582.1) was used for relative gene expression normalization. In addition, RT-qPCR data were analyzed using the Origin 8 software. As shown in Fig. 6, there were strong positive correlations (R^2^ = 0.98–0.96) between RNA-seq data and qRT-PCR data, demonstrating the reliability of the RNA-seq data.

## Discussion

### Transcriptome analysis of SE initial dedifferentiation in two cotton cultivars

*Gossypium hirsutum* is one of the most recalcitrant cultivars for *in vitro* plant regeneration through somatic embryogenesis. Moreover, only a few cultivars can produce SE. The number of cotton totipotent cells differentiating into calli is key to manipulating the SE process. Despite a few studies conducted to determine the molecular mechanisms regulating SE initial dedifferentiation^16^, different cultivars for SE initial dedifferentiation have been not well elucidated. In the present study, gene expression changes during SE initiation and formation were analyzed between the cultivars in which it was easy or difficult to produce SE. Our analyses showed that the number of DEGs and GO identified in similar types of cotton at different induction stages were generally higher than those observed during same-stage comparisons between HD and LD, indicating that HD and LD of different differentiation ability may be not only its varieties of differences, the more important be the response diversity for induction.

The DEGs and GO groups showed many differences in the 3h vs 0h and 3d vs 0h comparisons, indicating that 3h and 3d of induction may be the main regulation phase in the SE initiation. Previous studies have shown that hypocotyls cultured for 3h had no morphological changes compared to non-induction hypocotyls^16^. In tobacco and Arabidopsis, cellular dedifferentiation and initiation of cell division by protoplasts occurs within 48–72 h. Moreover, histological observations have shown that cotton somatic cells undergo initial differentiation within 72 h^19^. Furthermore, GO enrichment analysis revealed that protein kinase activity, oxidation-reduction processes and protein/ATP binding were associated with a higher number of DEGs in different and same time stages between HD and LD. Therefore, we inferred that these GO terms would be more likely to play a role in cells differentiating during the SE process.

### Stress-related transcription factors regulate differentiation of cotton SE

Transcription factors play regulatory roles in the embryogenesis processes in different plant species^20^. In the present study, 624 TFs were identified, among which the TF families involved in diverse stress responses as well as in SE processes such as *MYB*, *bHLH*, *bZIP* and *GRAS* are of particular interest^21, 22^. In *Arabidopsis* SE initiation, half of the stress-related transcription factors can be induced by 2, 4-D^23^. Stress and differentiation are strongly interrelated, overlapping processes^24^, with certain cells being competent to switch cell fate^25^. In our study, most of the TF families displayed diverse expression profiles that have complex regulatory functions during SE in cotton. Furthermore, the most differentially expressed TFs were identified in the greater the difference differentiation rate between the varieties.

In *Arabidopsis*, *bHLH* transcription factors play a pivotal role in cell fate determination^22^. *UPBEAT1* (*UPB1*), a *bHLH* transcription factor, regulated the balance between cell proliferation and differentiation by directly regulating the expression of a set of peroxidases^26^. Three *UPB1* homologues were expressed at higher levels at 3h, and the characterized *UPB1* might indicate that cell fate was changed from cellular proliferation to initial dedifferentiation at 3h. *MYC1* and *GL3* influence cell differentiation for root formation^27^. MYB proteins work together with BHLH proteins in a variety of cellular processes including control of cell fate determination and regulation of the cell cycle^28^. The *MYB* transcription factors, *CPC* and *ETC1*, are central regulators of cell differentiation in *Arabidopsis thaliana*, and *ETC1* has enhancer functions with *CPC* ^29, 30^. In the present study, the expression patterns of *CPC* and *ETC1* were consistent with those during three time stages. Finally, lower expression levels were observed in the *CPC* gene than in the two *ETC1* (Fig. 4b,c).

*GAI* is a highly homologous DELLA protein^31^. DELLA proteins are also GA signaling repressors that are responsive to GA-induced degradation by the ubiquitin-proteasome pathway^32^, as well as activation of GA-mediated physiological and developmental processes^33^. GA is a negative regulator of SE in Arabidopsis^34^. As revealed in this study, *GAI* decreased with increasing induction times. In plants, the *bZIP* transcription factors regulate diverse functions, such as plant development and stress response^35^. In the present study, *bZIP1*, *ABF2* and *ABF3* showed the same expression pattern. *GmbZIP1* was most closely related to *AtABF2* ^36^. Cross-talk between *bZIP1* and ABA occurred, potentially leading to improved tolerance to abiotic stresses^36^. In contrast, *GmbZIP44* is a negative regulator of ABA signaling^37^ that showed the opposite expression model compared to *bZIP1*. In the present study, *bZIP* homologues showed diverse expression profiles, which suggests a complex regulated by *bZIP* proteins as an ABA-dependent transcription factor in cotton SE. In addition, low ABA:GA ratios acted synergistically to stimulate SE in *Medicago truncatul*a^34^. Our results suggest that these TFs interactions and crosstalk regulate stress response and influence the initiation of SE differentiation. However, further research is required to determine how TFs affect differentiation of cotton SE.

### Reactive oxygen species (ROS) regulated differentiation of SE initiation

**Figure 7:**
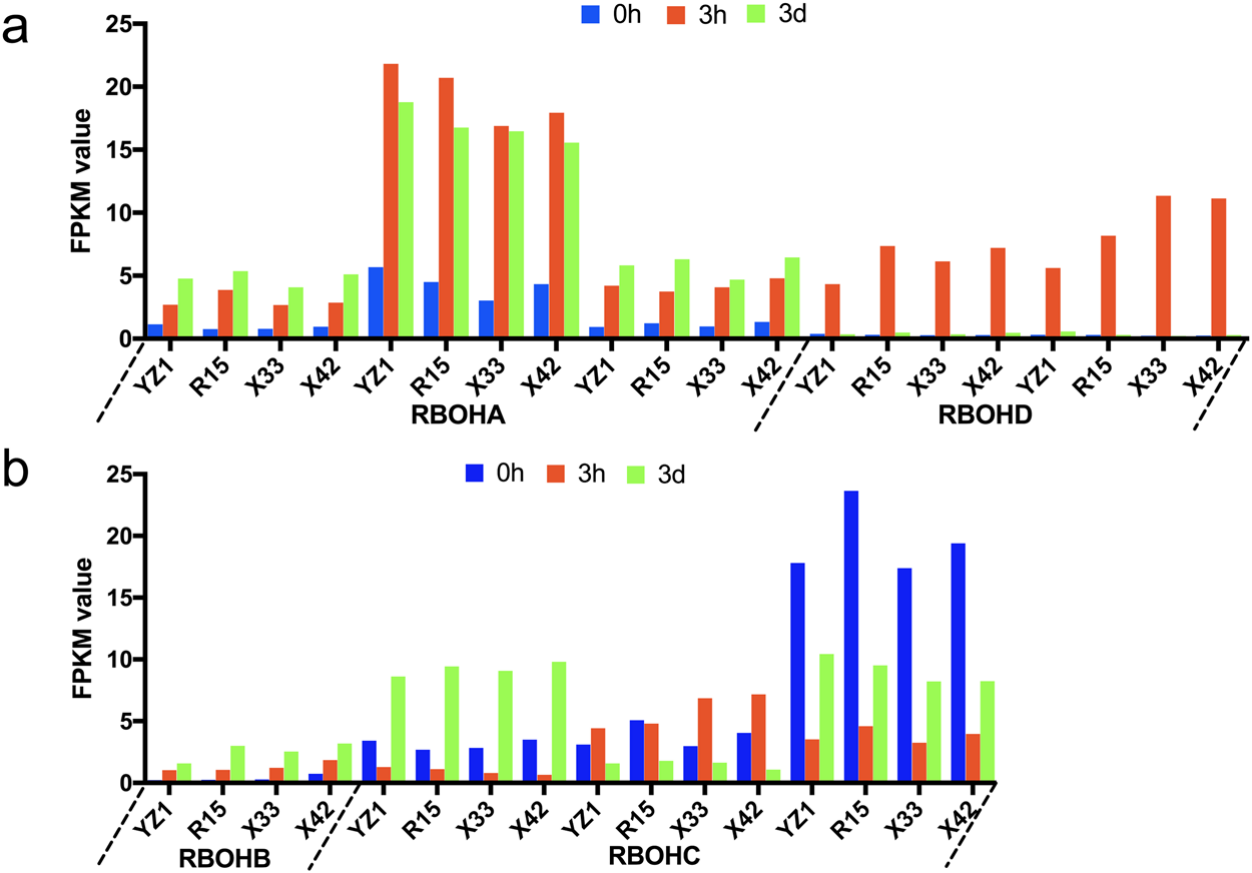
Expression of *ROS* genes.

According to our transcriptomic data, the oxidation-reduction process was significantly enriched in most comparisons. Redox homeostasis is essential to sustain metabolism and growth. In this study, some oxidative phosphorylation related genes also showed differential expression. The strength and duration of signaling regulated reactive oxygen species (*ROS*) via the redox-dependent signal transduction pathway^38^. *NADPH* oxidase (RBOH protein) is an important generator of ROS in the *M. truncatula* SE induction period^34^. The major *NADPH* oxidase catalytic subunits of homologues *AtRBOHD* and *AtRBOHF* are required for accumulation of ROS intermediates in the plant defense response in Arabidopsis^39^. In our study, the same expression model was observed in two *RBOHD* homologous genes, with relatively high expression occurring at 3h compared to 0h and 3d (Fig. 7a,b). In addition, *RBOHA*, *RBOHB* and *RBOHC* were found to have different expression patterns. Most studies of the interaction between ROS and other genes have been well documented. *AtRBOHD* and *AtrRBOHF* mediate ABA-induced ROS production in *Arabidopsis* ^39^. The *UPBEAT1* gene of the *bHLH* TF family controls the transition from cellular proliferation to differentiation by directly regulating the expression of ROS^26^. *DELLA* activity is regulated by environmental variability^40^, and *DELLAs* then regulate plant growth and stress tolerance through modulation of ROS levels^40^. Our results suggest that synergistic interactions between ROS and other genes may be essential for the differentiation of SE initialization.

### Crosstalk among auxin and ethylene contributes to initiation of SE differentiation

**Figure 8:**
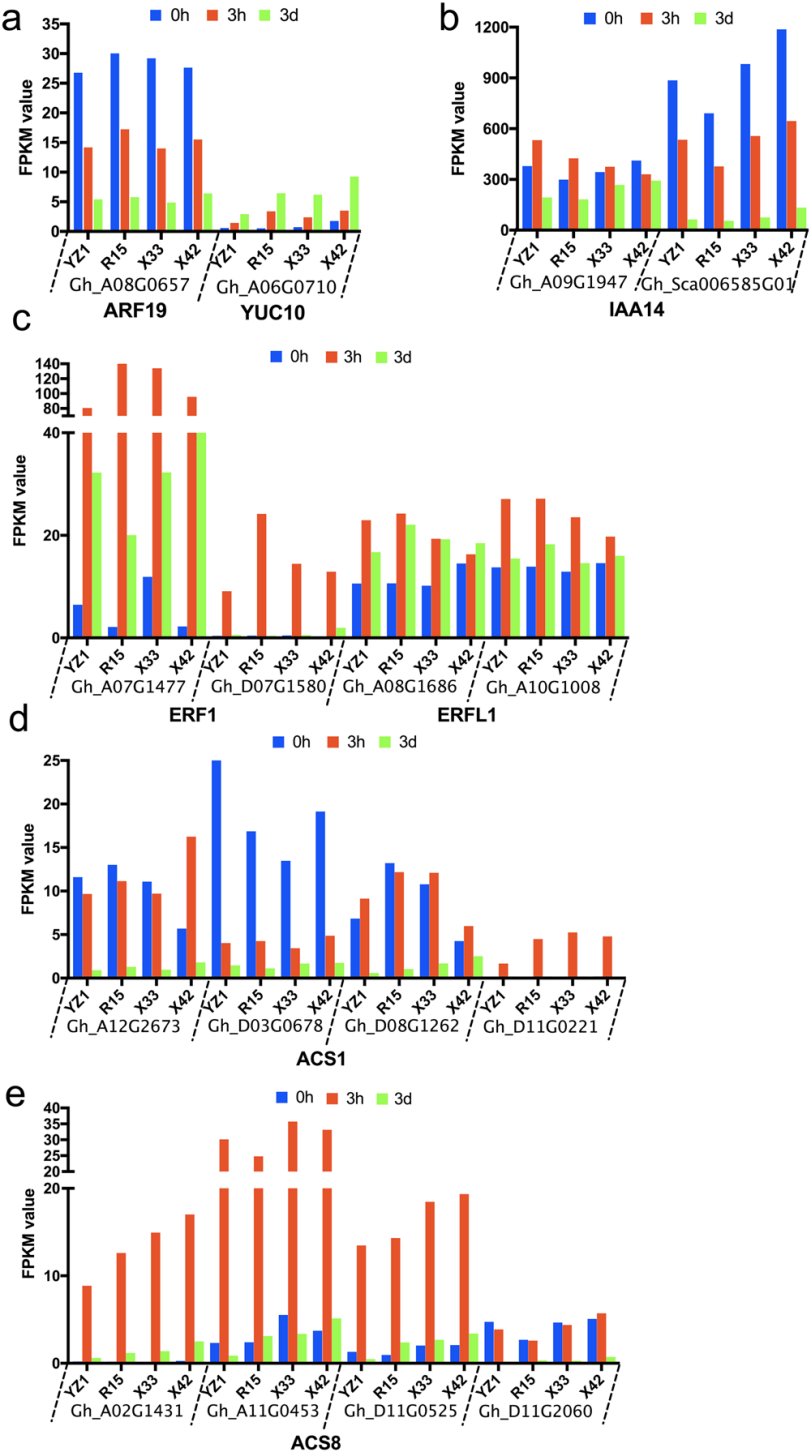
Expression of auxin (**a**–**c**) and ethylene (**d**–**h**) related genes at three time stages, 0h, 3h and 3d, in the two cultivars, HD and LD.

Auxin and ethylene are critical plant growth regulators (PGRs) for initiating differentiation in the induction of SE. The differentiation of cotton is correlated with sharp changes in endogenous anxin levels, which may be one of the first steps regulating SE^41^. *ARFs* and *AUX/IAAs* are the key transcription factors involved in regulating the expression of auxin-responsive genes. *ARFs* bind auxin response promoter elements, mediate transcriptional responses to auxin and regulate auxin-mediated transcriptional activation/repression together with *Aux/IAA* ^42^. *ARF19* and *ARF* TF family members^43, 44^ were down-regulated during two induction stages (Fig. 8a). The function of *ARF19* may be negatively regulated by *IAA14* and other *Aux/IAAs* ^44^. In addition, auxin induces degradation of *Aux/IAAs*, which release *ARFs* to regulate transcription of their target genes, thus establishing a negative feedback loop. In the present study, two *IAA14* (Fig. 8b) displayed different expression patterns, with one (Gh_Sca006585G01) showing the same pattern as *ARF19*. Gh_A09G1947 was up-regulated at 3h, then down-regulated at 3d. *YUCCA (YUC)* is an auxin biosynthetic gene^45^, and *YUC10* is essential for embryogenesis and leaf formation in *Arabidopsis* ^45^. In the present study, *YUC10* increased continuously during the three time stages (Fig. 8a).

*AP2-EREBP* is crucial for ethylene-regulated developmental processes, and ethylene may play a positive role in SE. *ERFs* contain an AP2 DNA-binding domain of transcriptional factors^46^. *ERF1* (*ETHYLENE RESPONSE FACTOR1*) is a key integrator of the ethylene responses in developmental processes^47^. In our study, two *ERF1* and two *ERFL1* were identified (Fig. 8c), with the highest expression level being observed in 3h. Ethylene also interferes with auxin during the initiation of embryogenesis^1^. Previous research showed that auxin could increase ethylene levels via activation of the expression of *ACC* (1-aminocyclopropane-1-carboxylic acid) synthase genes, leading to induction of the expression of *ARF19* to regulate downstream gene expression. *ARF19* serves as a cross talk point between auxin and ethylene^43^. In the present study, the FPKM of *ACS1* and *ACS8* showed the lowest level at 3d (Fig. 8d,e). Both auxin related genes and ethylene related genes were differentially expressed in the two cotton cultivars. Overall, auxin related genes and ethylene related genes showed complex expression profiles, and the results indicate that they may crosstalk with each other during SE dedifferentiation in different cultivars.

### Complex regulation of LRR-RLKs in cotton SE initiation

In the present study, many genes were classified into “protein kinase” and “protein/ATP binding” terms, such as LRR-RLKs (leucine-rich receptor-like protein kinase family protein), which have been shown to play important roles in cell differentiation and embryo pattern formation^48^. *SERKs*, *TDR/PXY* and *BAM1* belong to the LRR-RLK family of proteins. In *Arabidopsis*, *BAM1* plays an important regulatory role in stem cell maintenance^49^. In the present study, *BAM1* showed significant differences at 3h vs 0h and 3d vs 0h (Table 3). *SERK* genes play an essential role in determining embryogenic competence. In Arabidopsis, *SERK* proteins included two distinct groups, one group containing *SERK1* and *SERK2*, *BAK1*, *SERK4*, and *SERK5* are clustered to another group^50^. Our results showed that *SERK1*, *SERK2*, *BAK1* and *SERK1* displayed similar expression patterns between HD and LD, while *SERK2* and *BAK1* expression differed significantly in LD at 3h vs 0h. *BAK1* cannot replace *SERK1* in male fertility^50^. *TDR/PXY* also belongs to the LRR-RLK subclass^51^. *SERKs* serve as co-receptors in *TDIF* that interact with *PXY* to regulate plant development^51^. *CLE* (*CLAVATA3/EMBRYO SURROUNDING REGION-RELATED*) contains a conserved *CLE* domain at the C-terminal region and a hydrophobic signal peptide at the N-terminal region (*CLAVATA3/EMBRYO*)^52, 53^. Based on bioactivity and receptor specificity, R-type CLE and H-type CLE can be recognized in the CLE peptide family^54^. *CLV3* (*CLAVATA3*) belongs to R-type CLE, while the H-type CLE includes *TDIF*, and *CLE* peptides play important roles in regulating differentiation of stem cells and maintenance of shoot apical meristems in *Arabidopsis* ^52, 55, 56^. When compared to HD and LD, CLE proteins showed more differences in LD species at different time stages. When combined with the results of previous studies, our results indicate that *LRR-RLKs* play a role in the SE process and regulate the SE differentiation rate.

## Materials and Methods

### Plant materials and tissue culture conditions

Four cultivars of *Gossypium hirsutum*, YZ1, R15, X33 and X42, were investigated, among which YZ1 and R15 have a relatively high SE differentiation rate and therefore comprise the main transgenic material^57^. Although X33 and X42 are the major commercial cultivars in Xinjiang, China, they have a low rate of differentiation during SE compared with YZ1 and R15. Specimens of all cultivars have been conserved in our laboratory.

Four cotton seeds were sterilized with 0.1% (w/v) Hg_2_Cl_2_ for 10 min, then rinsed five times with sterilized distilled water. The sterilized seeds were germinated on ½ MS for hypocotyl induction^58^, then incubated at 28 °C in the dark for 6 d. Next, hypocotyls were excised from sterile seedlings, cut into 1 cm segments and placed on callus-induction medium (CIM; MS medium plus B5 vitamins, supplemented with 0.05 mg l^–1^ IAA, 0.05 mg l^–1^ kinetin, 0.05 mg l^–1^ 2,4-D, pH 5.8)^14^. Induction cultures were conducted under 16 h light: 8 h dark conditions at 28 °C. Different stages of explants at 0h, 3h and 3d were used for transcription analysis.

### RNA and library preparation for transcriptome sequencing

Total RNA was extracted from each sample using the PurelinkTM RNA Mini Kit (Life Technologies, Carlsbad, CA, USA) following the manufacturer’s protocol. We collected 24 samples from two biological replicates of each sample and monitored RNA degradation and contamination on 1% agarose gels. The RNA purity was checked using a NanoPhotometer^®^ spectrophotometer (IMPLEN, CA, USA), while the RNA concentration and integrity were measured using a Qubit® RNA Assay Kit and a Qubit® 2.0 Flurometer (Life Technologies, CA, USA) and the RNA Nano 6000 Assay Kit of the Bioanalyzer 2100 system (Agilent Technologies, CA, USA). RNA samples were stored at −80 °C until further processing. For each sample, a total of 3 µg RNA was used as the input material for the RNA sample preparations. Sequencing libraries were generated using NEBNext® Ultra™ RNA Library Prep Kits for Illumina® (NEB, USA) according to the manufacturer’s instructions, and index codes were added to attribute sequences to each sample. Clustering of the index-coded samples was performed on a cBot Cluster Generation System using the TruSeq PE Cluster Kit v3-cBot-HS (Illumia) according to the manufacturer’s recommendations. Finally, paired-end reads of 125 bp/150 bp were generated via an Illumina Hiseq platform.

### Bioinformatics analysis of RNA-Seq data

Quality control is first step, clean reads were obtained by removing adaptor tags, reads containing ploy-N and low quality reads from the raw reads of fastq format. In the addition, the clean reads of Q20, Q30 and GC were calculated, and high quality clean reads were used in all downstream analyses. For annotation, all reads were mapped to the reference sequences. An index of the reference genome was constructed using Bowtie v2.2.3 and paired-end clean reads were aligned to the reference genome using TopHat v2.0.12. To quantify the gene expression level, we employed HTSeq v0.6.1 to calculate the numbers of mapped reads and then normalized the results to the FPKM (expected number of Fragments Per Kilobase of transcript sequence per millions of base pairs sequenced), which is the most commonly used normalized method for estimating gene expression levels that considers the effects of sequencing depth and gene length for the reads at the same time^59^. We used the DESeq R package (1.18.0) to confirm the differential expression analysis. The resulting P-values were adjusted using Benjamini and Hochberg’s approach for controlling the false discovery rate. We used a threshold value of an adjusted P-value (padj) ≤0.05 and a log2FoldChange ≥ 2 in at least one of these stages.

### Functional classification of differentially expressed genes (DEGs)

Functional analysis of DEGs including Gene Ontology (GO) and KEGG was performed. GO enrichment analysis of DEGs was implemented by the GOseq R package. We used the KOBAS software to test the statistical enrichment of differential expression genes in the KEGG database. GO terms and KEGG pathways with corrected P-values ≤ 0.05 were considered the thresholds to determine significant enrichment by differentially expressed genes.

### qRT-PCR analysis

Eleven DEGs were selected to estimate the validation of RNA-seq data by qRT-PCR. Gene primers (Table S2) were designed using NCBI/Primer-BLAST (https://www.ncbi.nlm.nih.gov/tools/primer-blast/index.cgi?LINK_LOC=BlastHome) and synthesized by BGITECH. The cDNA was synthesized from 1 μL of total RNA (200–500 ng) using the PrimerScript RT reagent Kit (Takara, Dalian, China) in a 10 μL reaction mixture according to the manufacturer’s instructions. qRT-PCR was performed in 10 μL reactions on a LightCycler^®^ 480 (Roche) using 1 μL of first-strand cDNA as the template, 5 μL of 2 × SYBR Premix Ex Taq II (TLi RanseH Plus) (Takara, Dalian, China), 0.4 μL each of 10 μM forward and reverse gene-specific primers and 3.2 μL of ddH2O in 96-well plates. The cotton *ERF1α* (NCBI Reference Sequence: XM_016892582.1) was used as an internal standard. The qRT-PCR conditions were as follows: pre-incubation at 95 °C for 30 s, followed by amplification by 45 cycles at 95 °C for 10 s, 60 °C for 10 s and 72 °C for 10 s. qRT-PCR analysis was conducted in the three biological replicates. Data were analyzed using the Origin 8 software.

## Electronic supplementary material

Supplementary Table1

Supplementary Table2

Supplementary Table legends
