# Supplementary material for: Comparative Transcriptome Analysis of SE initial dedifferentiation in cotton of different SE capability
Source: Sci Rep. 2017 Aug 17;7:8583. doi: 10.1038/s41598-017-08763-8 (PMC5561258; doi:10.1038/s41598-017-08763-8)
Supplement: Supplementary file 3 — Supplementary Table legends [file 41598_2017_8763_MOESM3_ESM.doc]

Comparative Transcriptome Analysis of SE initial dedifferentiation in cotton of different SE capability

*Aiping Cao**^1^, Yinying Zheng^2^, Yu Yu^3^, Xuwen Wang^3^, Dongnan Shao^1^,* *Jie Sun^1^* and Baiming Cui^2^**

*1 The Key Laboratory of Oasis Eco-Agriculture, Shihezi University, Shihezi, China*

*^2^ Colleges of Life Science, Shihezi University, Shihezi, China*

*3 Cotton research Institute, XinJiang Academy of Agricultural and Reclamation Science, Shihezi, China*

**Corresponding, baimingc@shz.edu.cn*

Supplementary Table S1. Summary of all sequence data.

Supplementary Table S2. qRT-PCR primers used in this study.
